# Spiral ganglion cells and macrophages initiate neuro-inflammation and scarring following cochlear implantation

**DOI:** 10.3389/fncel.2015.00303

**Published:** 2015-08-12

**Authors:** Esperanza Bas, Stefania Goncalves, Michelle Adams, Christine T. Dinh, Jose M. Bas, Thomas R. Van De Water, Adrien A. Eshraghi

**Affiliations:** Department of Otolaryngology, Miller School of Medicine, University of MiamiMiami, FL, USA

**Keywords:** neuro-inflammation, fibrosis, pathology, cochlea, cochlear implant, Schwann cells, macrophages, spiral ganglion neurons

## Abstract

Conservation of a patient's residual hearing and prevention of fibrous tissue/new bone formation around an electrode array are some of the major challenges in cochlear implant (CI) surgery. Although it is well-known that fibrotic tissue formation around the electrode array can interfere with hearing performance in implanted patients, and that associated intracochlear inflammation can initiate loss of residual hearing, little is known about the molecular and cellular mechanisms that promote this response in the cochlea. *In vitro* studies in neonatal rats and *in vivo* studies in adult mice were performed to gain insight into the pro-inflammatory, proliferative, and remodeling phases of pathological wound healing that occur in the cochlea following an electrode analog insertion. Resident Schwann cells (SC), macrophages, and fibroblasts had a prominent role in the inflammatory process in the cochlea. Leukocytes were recruited to the cochlea following insertion of a nylon filament in adult mice, where contributed to the inflammatory response. The reparative stages in wound healing are characterized by persistent neuro-inflammation of spiral ganglion neurons (SGN) and expression of regenerative monocytes/macrophages in the cochlea. Accordingly, genes involved in extracellular matrix (ECM) deposition and remodeling were up-regulated in implanted cochleae. Maturation of scar tissue occurs in the remodeling phase of wound healing in the cochlea. Similar to other damaged peripheral nerves, M2 macrophages and de-differentiated SC were observed in damaged cochleae and may play a role in cell survival and axonal regeneration. In conclusion, the insertion of an electrode analog into the cochlea is associated with robust early and chronic inflammatory responses characterized by recruitment of leukocytes and expression of pro-inflammatory cytokines that promote intracochlear fibrosis and loss of the auditory hair cells (HC) and SGN important for hearing after CI surgery.

## Introduction

Cell regeneration (Löwenheim et al., 1999; Stone and Rubel, 2000; Kawamoto et al., 2003; Levic et al., 2007; Chen et al., 2013; Mizutari et al., 2013; Shi et al., 2013) and stem cells (Ito et al., 2001; Li et al., 2003; Martinez-Monedero and Edge, 2007; Koehler et al., 2013; Bas et al., 2014) are emerging therapies that aim to restore hearing in patients with deafness. Despite promising results, these novel therapies will take a long time to reach clinical application due to concerns regarding both safety and efficacy. Cochlear implantation is still one of the best options for patients with unserviceable hearing.

Cochlear implantation can restore hearing perceptions in patients with significant sensorineural hearing loss (SNHL) by bypassing the auditory hair cells (HC) and directly stimulating the spiral ganglion neurons (SGN). Non-traumatic cochlear implantation can be associated with preservation of auditory HCs of the apical and middle turns of the cochlea, which are important for low and even mid-frequency residual hearing, respectively. Significant gains in hearing in quiet and in noise as well as improvements in music perception are associated with residual “acoustic” hearing preservation following cochlear implantation (Mowry et al., 2012). Another important factor associated with better hearing outcomes following CI surgery is preventing excessive cochlear fibrosis, because fibrosis in the cochlea can negatively impact electrode impedance and “electrical” hearing perception with CIs (Hughes et al., 2001; Choi and Oghalai, 2005; Jia et al., 2011; Wolfe et al., 2013; Mosca et al., 2014). Although there have been many triumphs in CI research, long-term residual hearing preservation and prevention of fibrous tissue formation around an electrode array are still major challenges in cochlear implantation (Santa Maria et al., 2013).

Hearing outcomes after CI surgery depend on the health of residual auditory HCs, SGNs, and the factors that support their survival. Auditory HCs and their supporting cells within the organ of Corti (OC) secrete neurotrophic factors that support and help maintain SGN viability (Santa Maria et al., 2014). In addition, the health of Schwann cells (SC, glial cells of the peripheral nervous system) that reside within the SGN fibers is also an important component of hearing after implantation. SCs are responsible for myelination of type 1 SGNs, assuring an insulating sheath around spiral ganglia axons for rapid propagation of action potentials from the cochlea to the cochlear nucleus within the central nervous system (Romand and Romand, 1990). Similar to auditory HCs and supporting cells of the OC, SCs also cross-communicate with SGNs through the expression of neurotrophins and their receptors to promote SGN homeostasis (Hansen et al., 2001). A loss of sensory auditory HCs and SCs can reduce the number and activity of afferent SGNs, which are crucial for hearing in cochlear implantation (Roehm and Hansen, 2005). Therefore, traumatic cochlear implantations can magnify post-operative electrical-acoustic hearing impairments through synergistic losses of auditory HCs and SGNs. The application of “soft” atraumatic surgical techniques in CI surgery and the development of electrode arrays designed to reduce friction and trauma in the cochlea can reduce auditory HC and SGN losses, thereby improving post-operative “electrical-acoustic” hearing outcomes (Coco et al., 2007; Bas et al., 2012a; Mowry et al., 2012).

Electrode array insertions into the cochlea can initiate loss of auditory HCs and SGN fibers through direct mechanical injury and expression of intracochlear inflammatory cascades that are detrimental to their survival. Through these mechanisms, an aberrant wound healing response is activated in the cochlea that leads to fibrosis. By understanding the linkages between electrode insertion trauma (EIT), inflammation, and fibrosis formation, therapeutic strategies can be developed against these signaling pathways to prevent auditory HC death, loss of SGNs, and fibrotic deposition to improve post-implantation hearing outcomes.

Using adult mouse *in vivo* and neonatal rat *in vitro* models of electrode analog insertion trauma (EIT) (Bas et al., 2012b), the molecular and cellular mechanisms involved in the inflammatory, proliferative and remodeling phases of wound healing within the cochlea and their role in fibrosis were investigated. The early inflammatory response characterized by inflammatory cell infiltration was studied using the *in vitro* model. An *in vivo* model was used to investigate both early and late phases of the inflammatory response as well as contributions of the proliferative and remodeling phases of pathological wound healing to fibrosis and scar formation after EIT. In summary, a robust neuro-inflammatory response occurs after EIT, which leads to impressive amounts of cell proliferation, tissue remodeling, and fibrosis in the cochlea *in vitro* and *in vivo*.

## Materials and methods

### Animals

For the *in vitro* section, 3 or 4 day old (P3–P4) Sprague Dawley rat pups were used (Charles River Laboratories, Wilmington, MA, USA). For the *in vivo* studies 1.5–2 month old Balb/c mice of either sex were used (The Jackson Laboratory, Bar Harbor, ME). The mice were housed in sterile cages in a Virus Antigen Free facility from the Division of Veterinary Resources of University of Miami and were fed sterilized standard diet and water *ad libitum*.

### In vitro

P3–P4 rat pups are anesthetized with ice for 15 min and then decapitated. The otic capsules were dissected using a surgical microscope and placed in cold and sterile phosphate saline buffer (PBS). Cochleae were randomly assigned to each experimental group: control (no EIT and no treatment), EIT, and EIT + dexamethasone (DXM) treatment. To simulate EIT in experimental cochleae, a 0.2 mm diameter monofilament fishing line (Cajun Line; W.C. Bradley Co., OK, USA) was introduced three to four times through a small (~0.3 mm diameter) cochleostomy that was created with a sharpened #5 Dumont forcep next to the round window membrane. With this technique, a high angle (110–150°) and depth of insertion (2 mm) into the *scala tympani* was achieved (Bas et al., 2012b). All cochleae are then incubated for 10 min in PBS. Subsequently, whole OC with lateral wall tissues were harvested and cultured in serum-free culture media consisting of Dulbecco's modified Eagle's medium (DMEM, Invitrogen, Carlsbad, CA, USA) supplemented with glucose (final concentration at 6 g/L), 1% of N-1 supplement (Sigma Aldrich, St. Louis, MO, USA), penicillin G (30 U/mL), and either saline or DXM (20 μg/ml, D1756, Sigma Aldrich). The spleens of the pups were also harvested and kept in DMEM at 4°C. Leukocytes were isolated from spleen at 24 h and incubated in a culture dish for 2 h at 37°C. Supernatant was discarded and the adhered cells were collected. An aliquot of the cells was analyzed by Flow Cytometry (LSR-II, BD Biosciences, San Jose, CA) to confirm 90–95% enrichment in the monocytes population. To assess leukocyte recruitment and invasion into injured cochlear tissues, the monocytes were labeled with QTracker 655 (Life Technologies, Carlsbad, CA), re-suspended in PBS containing 2% FBS, incubated for 1 h at 37°C, and exposed to cochlear tissues (whose media was replaced for PBS + 2% FBS) prior to acquisition of images. For gene expression studies, the monocytes were re-suspended in serum free culture media and placed in inserts for indirect co-culture with the cochlear tissues. Both monocytes and cochlear tissues were collected at 72 h after co-culture, washed with cold PBS and stored in Trizol (Life Technologies, arlsbad, CA) at −80°C until further processing.

### Imaging and analysis of leukocyte behavior in the damaged cochlea microenvironment

Four cochlear tissue explants (i.e., OC with lateral wall tissues) were used for each condition with a total of 3 independent replicates. Conditions were control, EIT, and EIT + DXM. Sequential images of each group were taken every 15 s for 20 min with a 20 × lens in a Zeiss LSM 700 confocal upright microscope. ImageJ was used to analyze the images. Manual tracking (Manual Track plugin) was performed for 25–30 random cells in each sample. The trajectory and distance that the leukocytes traveled were measured. The numbers of leukocyte-leukocyte and leukocyte-tissue interactions were counted during this time period and the durations of these interactions were also documented in a double-blinded manner. The distance and trajectory of each cell was calculated as follow. $Distance=\sqrt{{\left({\text{X}}_{n}-{\text{X}}_{0}\right)}^{2}+{\left({\text{Y}}_{n}-{\text{Y}}_{0}\right)}^{2}}$, where (*x*_*n*_, *y*_*n*_) are the coordinates for the last time point (*n*) and (*x*_0_, *y*_0_) are the origin coordinates. $Trajectory=\sum_{0}^{n}\sqrt{{\left({\text{X}}_{i}-{\text{X}}_{\left(i-1\right)}\right)}^{2}+{\left({\text{Y}}_{i}-{\text{Y}}_{\left(i-1\right)}\right)}^{2}}$, where *X*_*i*_ = *X* − *X*_0_ and *Y*_*i*_ = *Y* − *Y*_0_ for each coordinate and (*X*_(*i*−1)_, *Y*_(*i*−1)_)are the coordinates for the previous time point. For display purposes, the TrackMate plugin was used. For display purposes, the TrackMate plugin was used. For leukocyte-leukocyte and leukocyte-cochlear tissue interaction counts and duration of the interactions, each sequence of images was divided in 4 quadrants (or regions of interest, 320 × 320 μm) and 1–80 cells were analyzed in each region.

### Gene expression analysis of the indirect co-cultured leukocytes and injured cochlear tissues

Six cochlear tissue explants (i.e., OC with lateral wall tissues) were used in each group and 4 independent replicates were done. Groups were control, EIT, and EIT + DXM. Leukocytes were removed from leukocyte-and-cochlear tissue co-cultures and analyzed separately. RNA was extracted with Trizol reagent (Invitrogen, Carlsbad, CA, USA) according to the manufacturer's protocol. RNA purity and concentration were determined by the absorbance at 260 and 280 nm using a Nano Drop ND-1000 (Thermo Fisher Scientific, Waltham, MA). iScript kit (Bio-Rad, Hercules, CA, USA) was used to synthesize the cDNA. Quantitative real-time PCR was performed in duplicate by using iQ SYBR Green Supermix (Bio-Rad) on a iCycler Real-Time CFX96 Detection System (Bio-Rad). The mRNA levels were normalized against β-actin (a housekeeping gene). The primers were designed based on the cDNA sequences obtained from Ensembl Genome Browser (http://www.ensembl.org).

The primers used were: Chemokine *Ccl2* (ENSRNOT00000009448, NM031530) forward 5′-TAATGCCCCACTCA CCTGCT-3′, reverse 5′-AGGTGCTGAAG TCCTTAGG-3′; *Sele*, (ENSRNOT00000076757, NM138879) forward 5′-GAGATCTAC ATCCAAAGACC-3′, reverse 5′-CTTTACATT CAACCACATGGC-3′; *Sell* (ENSRNOT00000003733, NM019177) forward 5′- CAGTGTCAGT ATGTGATCC-3′, and reverse 5′-GACATA TTGGACTAGGAC-3′; *Icam1*, (ENSRNOT00000028066, NM012967) forward 5′-CTGTGTATTCG TTCCCAGAGC-3′ and reverse 5′-GATCGAAA GTCCGGAGCT-3′; *Vcam1* (ENSRNOT00000019377, NM012889) forward 5′-GACATCTACTCATT CCCTAAGG-3′ and reverse 5′-GGAGGTG TAGACTTGTAGT-3′; *Il10* (ENSRNOT00000006246, NM012854) forward 5′-TAAGGGTTA CTTGGGTTGC-3′ and reverse 5′-CACCTTT GTCTTGGAGCTT-3′; *Tgfb1* (ENSRNOT00000028051, NM_021578) forward 5′-CGGACTACTAC GCCAAAGAA-3′ and reverse 5′-TCAAAAG ACAGCCACTCAGG-3′; *Actb* (ENSRNOT00000042459, NM031144) forward 5′-CGTTGACATCCGT AAAGACC-3′, and reverse 5′-AGCCACCAA TCCACACAGAG-3′ (Sigma Aldrich). Real-time PCR was performed using the following parameters: 3 min at 95°C followed by 40 cycles of 15 s at 95°C and 1 min at 60°C. Melting curves were also performed to ensure primer specificity and evaluate for any contamination. Relative changes in mRNA levels of genes were assessed using the ΔΔCt method and normalized to the house-keeping gene β-actin (*Actb*) and then to the expression levels of the control group.

### In vivo

Twenty adult mice were randomly assigned to each selected time point and group. The animals were anesthetized with a Ketamine (40 mg/kg)-Xylazine (5 mg/kg) cocktail. Buprinorphine (0.05 mg/kg) was administrated at this time and 2 times a day for 2 more days. The eye blink response to a light touching of the cornea and a toe pinch withdrawal response was used to determine the depth of the anesthesia, which was kept a surgical plane. We also looked at any changes in both rate and depth of inspirations in the breathing pattern of the anesthetized mice.

The post-auricular hair was shaved and the area cleaned with iodine. Artificial tears were added to each eye to prevent corneal dryness. Five microliter of 1% lidocaine was used for local anesthesia. A post auricular incision was performed behind the experimental ear and extended ventrally to the rostral neck skin. The subcutaneous connective tissues were separated to expose the deep structures. The ear canal, the sternocleidomastoid muscle and the facial nerve were identified. The bulla that encloses the middle and inner ears was visualized below the facial nerve. A self-retaining retractor was used to maintain exposure. The soft tissue structures overlying the temporal bone were removed to expose the bulla. A small hole was opened in the bulla and ~2 mm of sterile nylon monofilament (~0.2 mm diameter) was introduced through a puncture in the round window membrane and left in place. Tissue glue was used to plug the hole made into the bulla and the skin was sutured closed with nylon sutures and cleaned once more with topical iodine. The contralateral ear was used as an internal control. The animals were returned to clean cages fitted with a water circulating warming pad and checked periodically during the post-anesthesia recovery period. The animals were euthanized with inhaled CO_2_ at 1, 3, 7, 14, or 30 days after the implantation. The cochleae were kept either in Trizol at −80°C for mRNA processing or in freshly prepared 4% paraformaldehyde at 4°C for immunostaining processing.

### Genes involved in fibrosis

Cochleae from adult mice exposed to unilateral cochlear implantation for 7 days were utilized. Pooled samples from five adult mice non-implanted cochleae were compared to five implanted cochleae; experiments were replicated three times. The cochleae that were preserved at −80°C in Trizol (Life Sciences, Carlsbad, CA) were thawed in a bath of ice and homogenized. Total RNA was extracted following the manufacturer's protocol. The quantity and quality of RNA was measured. RT^2^ First Strand Kit for cDNA synthesis and RT^2^ SYBR Green qPCR Mastermix with a RT^2^ Profiler PCR Array for Mouse Fibrosis genes (all from Qiagen, Valencia, CA, USA) were used in the gene expression studies. Data analysis of the PCR Array was performed with Qiagen's web based software using the ΔΔCt method. The raw data was normalized to housekeeping genes (*Actb* and *Gusb*). Paired experiments were run and the average of the fold change between implanted cochleae and contralateral unoperated control was calculated.

### Immunohistology

Cochleae from mice exposed to unilateral cochlear implantation for 1, 3, 7, 14 and 30 days were utilized (*N* = 4 or 5 per condition). The cochleae kept in 4% in paraformaldehyde were transferred to 10% EDTA buffered in PBS at pH 6 and kept in gentle rotation for 7 days. The cochleae were washed in PBS and passed through a sucrose gradient from 5 to 30%. Cochleae were then frozen in O.C.T. compound media (Tissue-Tek, Sakura Finetek USA, Inc, Torrance, CA, USA) and cryosections were performed parallel to the central plane of the modiolus of the cochleae. Slides were washed in PBS and kept in a blocking-permeabilizating media (normal serum, 1% Triton x-100 in PBS) for 1 h. After this time, samples were incubated at 4°C overnight with either of the following primary antibodies: rabbit anti-Arginase I (sc-20150, Santa Cruz Biotech, Dallas, TX, USA), rabbit anti-Interleukin 1β pAb (sc-7884, Santa Cruz Biotech, Dallas, TX, USA) or rabbit anti-ITGA4 (A0696, Neobiolab, Woburn, MA, USA). The slides were washed 3 times with PBS and incubated for 90 min at room temperature with anti-F4/80-FITC (ab60343, Abcam, Cambridge, MA, USA) for Arg1 and IL-1β or Phalloidin-FITC (Sigma-Aldrich, St Louis, MO, USA), and the secondary antibody anti-rabbit IgG Alexa 633. After 3 more washes the specimens were stained with DAPI, washed and cover-slipped with anti-fade mounting media. The sections were observed under a Zeiss LSM 700/confocal upright microscope. Images of different areas of the cochlea were acquired and later constructed. ImageJ was used to analyze the images, histograms of red and green channels for each region of interest (i.e., lateral wall, organ of Corti, spiral ganglion, cochlear nerve, and wound site) were recorded.

### Histological distribution of the fibrotic tissue

Selected slides from cochleae cryosections were stained using a Masson's Trichrome Stain Kit (Polysciences, Inc, Warrington, PA, USA) following the manufacturer's instructions. Slides were then dehydrated in alcohol gradient, cleared with Histo-Clear (National Diagnostics, Charlotte, NC) and mounted with Cytoseal XYL (Richard-Allan Scientific, Campus Dr, Kalamazoo, MI). The specimens were observed under a Zeiss Axiovert 200 microscope with a × 10 lens and the mosaic images were stitched together afterwards from individual images.

### Statistics

One-Way ANOVA and Tukey's Multiple Comparison test were utilized for *in vitro* studies: leukocyte distance covered, trajectory of leukocytes, counts of leukocyte-leukocyte and leukocyte-tissue interactions, and duration of interactions. The same test was applied for the *in vitro* gene expression study. Two-Way ANOVA followed by Bonferroni *post-hoc* tests were used to analyze the relative and absolute fluorescence intensities from the adult cochleae immunolabeling studies (*in vivo* studies). In all graphs the results are expressed as mean values ± S.D. In the study of genes involved in fibrosis in adult mice cochleae, the results are expressed as mean values ± S.D. Genes from implanted cochleae that demonstrated differences of ≥2 mean fold changes over control values were considered up regulated. Mean fold changes from control values ≤0.5 were considered down-regulated.

## Results

### Increased leukocyte recruitment and cell-cell interaction in damaged cochlear tissues

**Figure 1:**
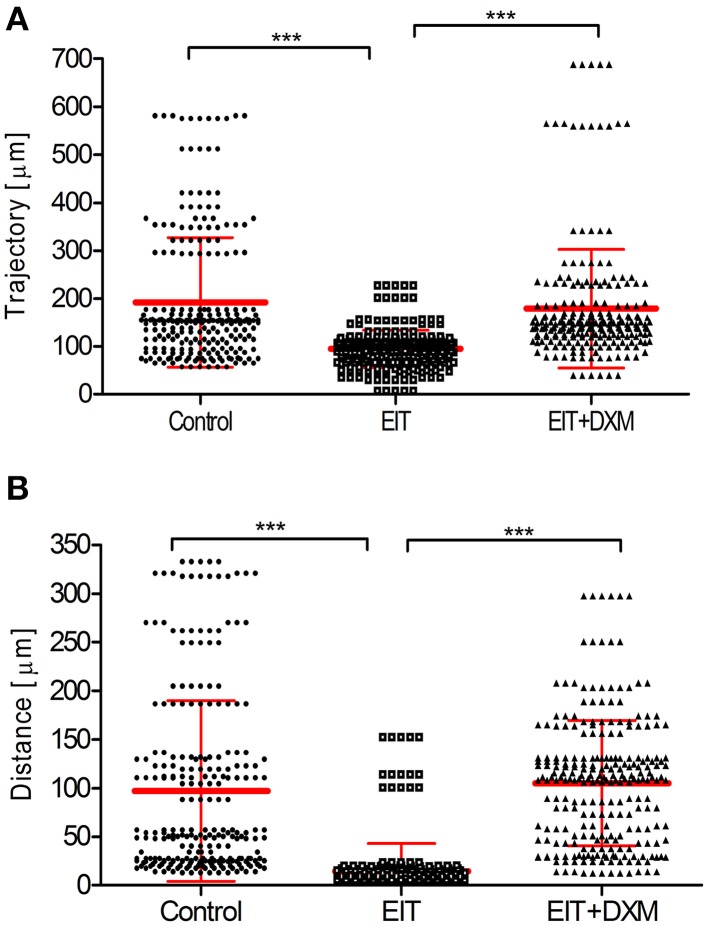
**Analysis of tracked cells for direct leukocyte and cochlear tissue co-cultures. (A)** Leukocyte trajectory and **(B)** leukocyte distance covered. Comparison are between EIT-cochlear tissues and undamaged controls and with EIT pre-treated with Dexamethasone (DXM, 20 μg/ml). *N* = 239−250 cells/group, One-Way ANOVA and Tukey's Multiple Comparison Test was used for statistical analysis ^***^p < 0.001.

Cell movement analyses: Figure 1 shows the analysis of tracked leukocytes exposed to EIT cochlear tissue explants. The trajectory (a, 94.7 ± 2.5, *N* = 250 cells, *p* < 0.001) and distance (b, 14.48 ± 1.8, *N* = 249 cells, *p* < 0.001) covered by these tracked leukocytes were significantly reduced compared with leukocytes co-cultured with undamaged control cochlear tissue explants (a, 191.8 ± 8.7, *N* = 239 cells; distance, b, 96.9 ± 5.9, *N* = 249 cells). The behavior of leukocytes was assessed in EIT cochlear tissue explants following DXM treatment (20 μg/ml), a synthetic steroid known to inhibit expression of pro-inflammatory cytokines, chemokines, and cell adhesion molecules in other tissue types. Leukocyte response in DXM treated EIT cochlear tissue explants was similar to responses observed in undamaged control explants and significantly different from EIT cochlear tissues (DXM treated: trajectory, a: 178.6 ± 8.0, *N* = 239 cells, *p* < 0.001 compared to EIT; distance, b: 105.2 ± 4.1, *N* = 249 cells, *p* < 0.001 compared to EIT). Videos of leukocyte recruitment and interaction in the three different groups of cochlear tissue explants can be viewed in the supplemental data section (Supplemental Movies 1–6).

**Figure 2:**
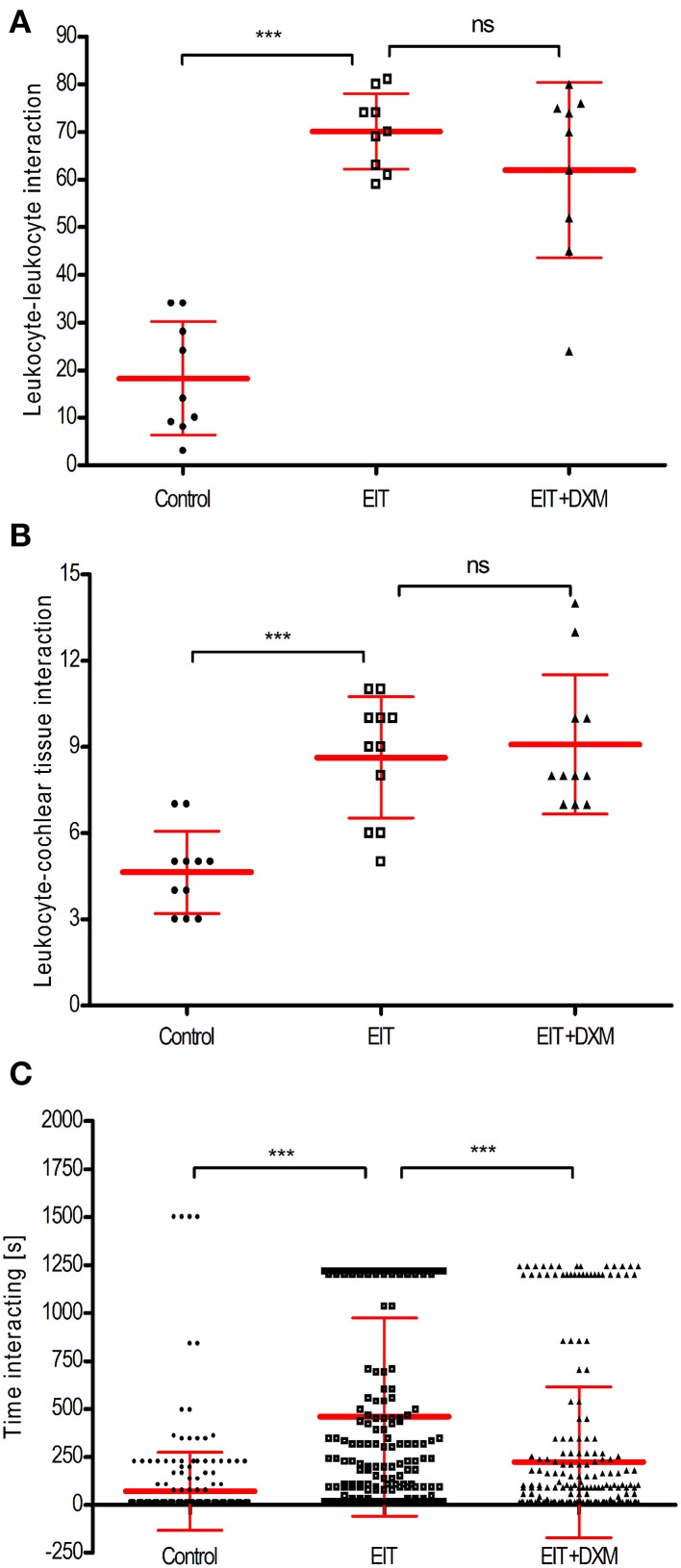
**Cell-cell interactions analysis for direct leukocyte and cochlear tissue co-cultures. (A)** Number of leukocyte-leukocyte interactions in leukocytes and cochlear tissues co-cultures; **(B)** number of leukocyte-cochlear cells interactions in co-cultures and **(C)** time average that leukocytes interact. Comparisons are between EIT-cochlear tissues and undamaged controls and with EIT pre-treated with dexamethasone (DXM, 20 μg/ml). In upper and middle graphs, the number of cells interacting was averaged from 9 to 11 quadrants or regions of interest. For the bottom panel, *N* = 278 cells/group. One-Way ANOVA and Tukey's Multiple Comparison Test was used for statistical analysis ^***^p < 0.001, ns non-significant *p*-value.

Each sequence of images was divided in quadrants or regions of interest (320 × 320 μm). We found a significant increase in the number of leukocyte-leukocyte (a, 70 ± 3, *N* = 9 regions of interest, *p* < 0.001) and leukocyte- cochlear tissue (b, 9 ± 1, *N* = 11 regions of interest, *p* < 0.001) interactions between the leukocytes co-cultured with the EIT explant group compared to the undamaged cochlear tissue explants (leukocyte-leukocyte, a: 18 ± 4, *N* = 9 regions of interest, and leukocyte-cochlear tissue, b: 5 ± 1, *N* = 11 regions of interest) (Figures 2A,B). DXM treatment did not affect the number of interactions between leukocytes and leukocyte-cochlear tissue explants, when compared to the EIT group of explants (DXM treated: leukocyte-leukocyte, a: 62 ± 6, *N* = 9 regions of interest, *p* > 0.05; leukocyte-cochlear tissue, b: 9 ± 1, *N* = 11 regions of interest, *p*>0.05 The duration of interactions between leukocytes in the EIT group of explants was significantly increased compared to values obtained in the undamaged control group of explants (458.20 ± 30.95 EIT vs. 71.76 ± 12.15 control explants, *N* = 278, *p* < 0.001) (Figure 2C). Interestingly, even though DXM did not alter the number of cell-cell interactions between leukocytes and leukocyte-and-cochlear tissue, the time that the leukocytes remained in contact was significantly shorter than durations observed in the EIT group of explants (c, DXM treated: 222.70 ± 23.53, *N* = 278).

### Cochlear tissues and leukocytes overexpress chemokines and cell adhesion molecules in response to an electrode analog insertion-induced trauma *in vitro*

**Figure 3:**
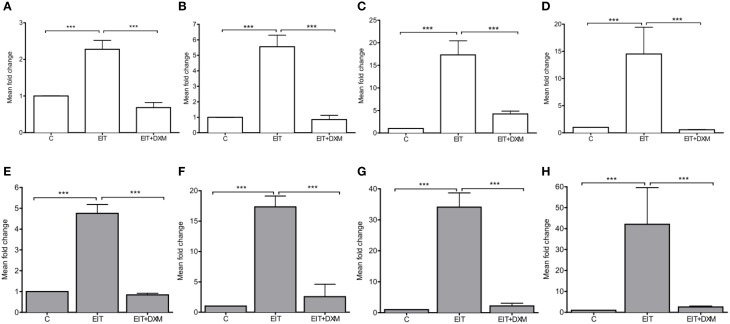
**Gene expression studies *in vitro***. The experiments were performed in indirect leukocyte and cochlear tissue co-cultures. The top panel shows the gene expression levels for leukocytes (white), where **(A)** *Ccl2*, **(B)** *Sell*, **(C)** *Icam*, and **(D)** *Tgfb1*. The bottom (gray) panel shows the gene expression levels in cochlear tissues, i.e., organ of Corti and lateral wall tissues, where **(E)** *Ccl2*, **(F)** *Sele*, **(G)** *Vcam*, and **(H)** *Tgfb1*. Comparisons are between EIT-cochlear tissues and undamaged controls and with EIT pre-treated with Dexamethasone (DXM, 20 μg/ml). Six animals per group were included in each set of experiments and a total of 4 independent experiments were carried out. *N* = 4, One-Way ANOVA and Tukey's Multiple Comparison Test was used for statistical analysis ^***^p < 0.001.

EIT injury in cochlear tissues is associated with increased mRNA levels for the potent chemokine *Ccl2* in both cochlear tissue (4.76 ± 0.21, *N* = 4, *p* < 0.001) and leukocytes that were co-cultured with EIT cochlear tissues (2.27 ± 0.12, *N* = 4, *p* < 0.001), when compared to mRNA levels for this chemokine in the undamaged control group (Figures 3A,B). A rise in the transcript levels that encode for the cell adhesion molecules *Vcam1* (34.11 ± 2.29, *N* = 4, *p* < 0.001) and *Sele* (17.34 ± 0.89, *N* = 4, *p* < 0.001) was observed in EIT cochlear tissues when compared to the undamaged control explants. Similarly, an increase in *Icam1* (17.31 ± 1.58, *N* = 4, *p* < 0.001) and *Sell* (5.55 ± 0.37, *N* = 4, *p* < 0.001) mRNA levels was observed in the co-cultured leukocytes from the EIT group of explants, when compared to uninjured explants. In addition, *Tgfb1*, a growth factor released by SCs and macrophages upon nerve injury, was overexpressed in both cochlear tissues (42.02 ± 8.77, *N* = 4, *p* < 0.001) and leukocytes (14.50 ± 2.46, *N* = 4, *p* < 0.001) in the EIT explant group, when compared to the uninjured control group. DXM treatment (20 μg/ml) of EIT cochlear tissues and leukocytes demonstrated significant reductions in *Ccl2, Vcam1, Sele, Icam1, Sell*, and *Tgfb1* gene expression levels when compared to EIT injured cochlear tissue and leukocytes (*p* < 0.05; Figure 3).

### Fibrosis-related gene expression levels *in vivo*

Real-time PCR: Gene expression profiling for the proliferative/fibrogenic process in cochlear tissue harvested from adult mice exposed to unilateral cochlear implantation for 7 days was performed (Table 1). In order to maintain consistency with the literature, genes with fold change = 2 in EIT cochlear tissue when compared to expression levels of contralateral un-operated cochleae were considered up-regulated. Gene expression levels ≤0.5 when compared to control cochleae were considered down-regulated. Consistent with previous publications of peripheral nerve injury and our *in vitro* gene expression data, there were increased expression levels of of Th1 (i.e., *Il1a* and *Il1b*) and Th-2 (i.e., *Il4* and *Il13*) types of cytokines in EIT cochlear tissues. The chemokines *Ccl3* and *Ccl12* responsible for the recruitment of leukocytes to the wound site were also up-regulated in implanted cochlear tissues. Higher levels of mRNA for platelet-derived growth factor beta polypeptide (*Pdgfb*), a protein that promotes proliferation, differentiation, and migration were observed in cochlear tissues traumatized by the monofilament insertion. Vascular endothelial growth factor A (*Vegfa*), which is a member of the PDGF family and promotes angiogenesis and vascular permeability, was also up-regulated in implanted tissues. mRNA levels for the integrin subunits α1 (*Itga1*), α2 (*Itga2*), α3 (*Itga3*), αv (*Itgav*), and β6 (*Itgb6*) were higher in EIT cochlear tissues when compared to contralateral ears, while *Itgb3* levels were lower in implanted cochleae.

**Table 1:** **Gene expression profile of the proliferative/fibrogenic process at 7 days post-cochlear implantation in adult mice**.

	**Mean fold**	**S.D.**		**Mean fold**	**S.D.**
*Mmp3*	35.80	10.38	*Snai1*	1.79	0.27
*Ccl12*	31.52	7.97	*Ltbp1*	1.75	0.35
*Timp1*	17.25	5.25	*Il5*	1.62	0.17
*Col3a1*	15.97	8.82	*Dcn*	1.61	0.31
*Ccl3*	12.74	4.71	*Tnf*	1.59	0.19
*Col1a2*	8.50	2.70	*Itgb8*	1.56	0.27
*Il13*	8.32	3.00	*Ccr2*	1.51	0.14
*Il13ra2*	6.04	1.41	*Edn1*	1.42	0.47
*Serpine1*	5.50	3.19	*Stat6*	1.42	0.43
*Mmp14*	5.21	2.93	*Stat1*	1.38	0.18
*Il1a*	5.12	0.83	*Smad2*	1.37	0.13
*Fasl*	4.95	1.32	*Timp3*	1.33	0.41
*Itgb6*	4.93	3.33	*Nfkb1*	1.31	0.20
*Il1b*	4.05	0.65	*Cebpb*	1.30	0.63
*Il4*	4.05	1.40	*Agt*	1.28	0.66
*Timp2*	3.89	0.78	*Eng*	1.25	0.46
*Thbs2*	3.75	1.35	*Smad6*	1.25	0.63
*Jun*	3.67	0.45	*Ccl11*	1.20	0.23
*Lox*	3.40	1.33	*Tgif1*	1.19	0.13
*Mmp13*	3.19	1.30	*Serpina1a*	1.19	0.21
*Serpinh1*	2.92	1.75	*Tgfbr1*	1.18	0.21
*Hgf*	2.79	1.11	*Smad4*	1.16	0.14
*Itga2*	2.73	0.55	*Plat*	1.15	0.18
*Plau*	2.58	1.39	*Cxcr4*	1.09	0.25
*Ctgf*	2.45	0.75	*Smad3*	1.08	0.28
*Itgav*	2.38	0.88	*Myc*	1.02	0.20
*Mmp2*	2.33	1.13	*Akt1*	1.00	0.32
*Itga3*	2.29	1.16	*Timp4*	0.99	0.21
*Vegfa*	2.22	0.69	*Thbs1*	0.97	0.19
*Itga1*	2.14	0.99	*Tgfb2*	0.95	0.17
*Acta2*	2.12	0.81	*Egf*	0.93	0.27
*Grem1*	2.11	1.41	*Itgb5*	0.93	0.35
*Tgfb1*	2.03	0.28	*Pdgfa*	0.81	0.11
*Pdgfb*	2.01	0.95	*Sp1*	0.81	0.08
*Itgb1*	1.95	0.25	*Smad7*	0.79	0.30
*Bmp7*	1.93	0.54	*Ilk*	0.71	0.07
*Mmp9*	1.81	0.51	*Mmp8*	0.64	0.09
*Cav1*	1.80	0.32	*Mmp1a*	0.63	0.18
*Ifng*	1.79	0.27	*Itgb3*	0.62	0.06
*Il10*	1.79	0.27	*Bcl2*	0.50	0.02
*Inhbe*	1.79	0.27	*Tgfb3*	0.50	0.06
*Plg*	1.79	0.27	*Tgfbr2*	0.47	0.10

Similar to the *in vitro* study results, *Tgfb1* gene expression levels were also higher in the adult implanted cochleae compared to the contralateral control cochleae at 7 days. Interestingly, *Tgfb3* and its receptor *Tgfbr2*, which are associated with scar-less wound healing were down-regulated in EIT cochlear tissues. mRNA expression levels for enzymes involved in extracellular matrix (ECM) remodeling such as *Mmp2, Mmp3, Mmp13, Mmp14*, Urokinase-type plasminogen activator (*Plau*), *Serpine1, Serpinh1, Timp1, and Timp2* were all increased following EIT, relative to un-implanted cochlear tissues. High gene expression levels for hepatocyte growth factor (*Hgf*, a protein involved in angiogenesis and tissue regeneration) and ECM components smooth muscle α-2 actin (*Acta2*), collagen type 1 (*Col1a2*) and 3 (*Col3a1*) were observed in traumatized cochleae when normalized to control cochleae. Lysyl oxidase (*Lox*, a protein coding gene that is known to mediate the cross-linking of the ECM proteins collagen and elastin), thrombospondin-2 (*Thbs2*; mediator of cell-cell and cell-matrix interactions), Gremlin-1 [*Grem1*, an antagonist of the bone morphogenetic proteins (BMP)] and *Jun* (proto-oncogene) were all over-expressed in cochlear tissues traumatized by EIT, when compared to the unoperated control group. In injured cochleae, Fas ligand (*Fasl*; initiator of the extrinsic pathway of cell death) was upregulated and *Bcl2* (anti-apoptotic protein involved in mitochondrial cell death) levels were significantly reduced relative to control cochleae.

### Early expression of integrin 4α following an electrode analog insertion trauma

**Figure 4:**
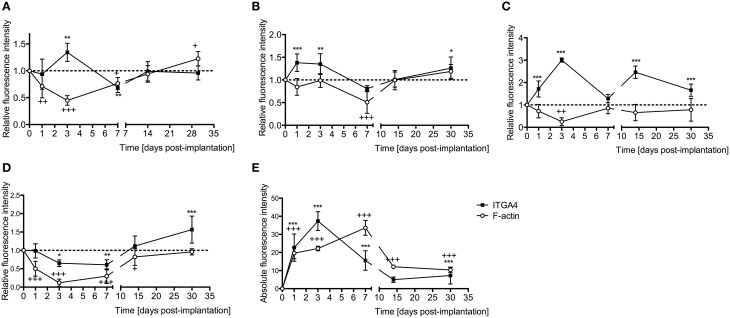
**Time-course immunofluorescence analysis for integrin 4α and f-actin levels in different areas of adult mice implanted cochleae. (A)** Lateral wall tissues (see Figure 6, spiral ligament, SL), **(B)** organ of Corti, **(C)** spiral ganglion, **(D)** cochlear nerve, and **(E)** wound site. Three to four specimens for each time point and were analyzed. ImageJ was used to analyze the images, histograms of red and green channels for each region of interest i.e., lateral wall, organ of Corti, spiral ganglion, cochlear nerve, and wound site images were recorded. *N* = 8 each time point, Two-Way ANOVA followed by Bonferroni posttests was used to analyze the relative and absolute fluorescence intensities. ^***^p < 0.001, ^**^p < 0.01, ^*^p < 0.05 and +++*p* < 0.001, ++*p* < 0.01, +*p* < 0.05 represent the *p*-values for ITGA4 and F-actin, respectively.

**Figure 5:**
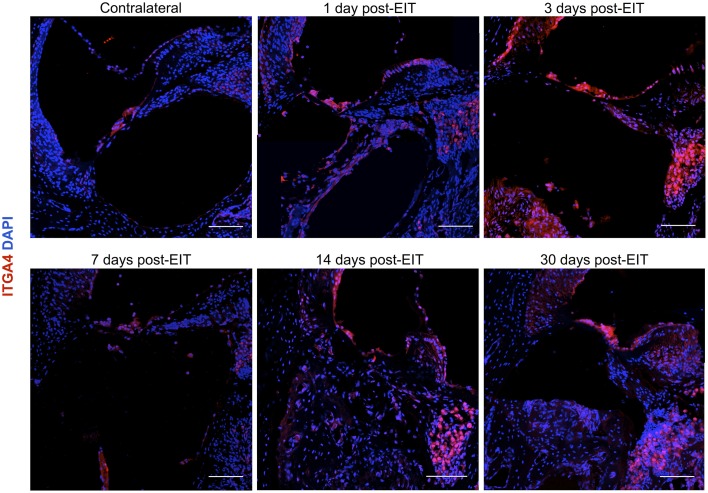
**Immunofluorescence images for integrin α4 (ITGA4) in red and cell nuclei in blue**. Cross sections corresponding to a contralateral cochlea (bottom), and cochleae at 1, 3, 7, 14, and 30 days post-implantation. The sections were observed under a confocal microscope. Mosaic images of different areas of the cochlea were acquired and later constructed. Three to four specimens were analyzed for each time point. Scale bars = 100 μm.

**Figure 6:**
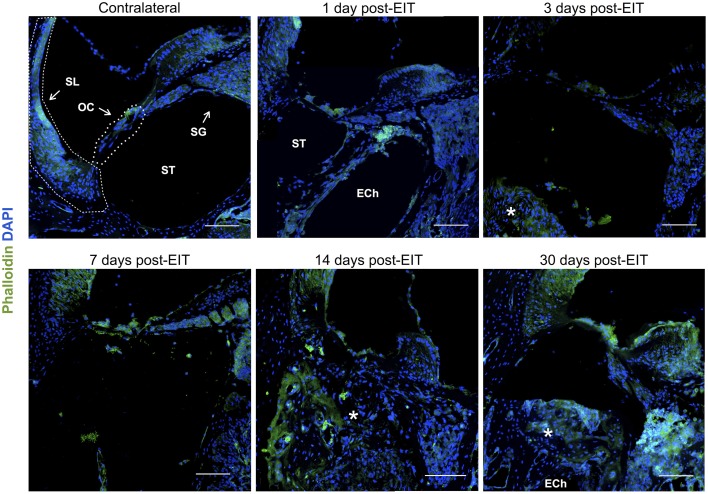
**Immunofluorescence images for f-actin in green and cell nuclei in blue**. Cross sections corresponding to a contralateral cochlea (bottom), and cochleae at 1, 3, 7, 14, and 30 days post-implantation. The sections were observed under a confocal microscope. Mosaic images of different areas of the cochlea were acquired and later constructed. Annotations for the different structures of the cochlea are in the contralateral photograph. Spiral ligament, SL; organ of Corti, OC; Scala tympanica, ST; Spiral ganglion, SG. In the implanted cochleae: electrode analog channel, ECh; an asterisk marks hyperproliferative tissue in the Scala tympanica. Three to four specimens were analyzed for each time point. Scale bars = 100 μm.

Integrin 4α (ITGA4) is a receptor that is expressed after peripheral nerve injury, binds to fibronectin, and is important for neuron regeneration and cell-matrix interactions during leukocyte recruitment. Fluorescence intensities were measured in cross sections of cochleae at 1, 3, 7, 14, and 30 days after EIT after immunofluorescence labeling for ITGA4. Relative fluorescence intensity values were obtained by normalizing data to contralateral un-operated cochleae of these same areas (Figures 4–6). There was increased ITGA4 expression at 1 and 3 days post-implantation, which rapidly dropped at 7 days in lateral wall tissues, OC, spiral ganglion, and site of monofilament insertion. ITGA4 expression either returned to baseline (lateral wall and OC), decreased (site of monofilament insertion), or increased (spiral ganglia) 14 days after implantation (Figures 4, 5).

Cell-cell junctions are comprised of cadherin, catenins, and filamentous actin (f-actin) cytoskeleton. Together, f-actin and myosin II form stress fibers, which are contractile bundles important for cell adhesion; they are abundant in endothelial cells, epithelial cells, and myofibroblasts. As a result, f-actin remodeling can affect the integrity of cell-cell junctions and destabilize the epithelial barrier, rendering leukocytes access to surrounding tissues. Reorganization of the actin cytoskeleton and loss of cell-cell adhesions are also seen in epithelial-mesenchymal transition, a phenomenon that occurs in fibrosis and wound healing (Haynes et al., 2011). A significant reduction in f-actin levels in lateral wall, spiral ganglion and cochlear nerve at 3–7 days *in vivo* contrasts with an increase on this stress fibers component in the area where the electrode-analog was placed (Figures 4, 6).

### Macrophages and Schwann cells involvement in early and chronic inflammatory responses

Glial cells are non-neuronal cells that are important for myelin formation. They also surround neurons, ground them to the ECM, supply nutrients to neurons, and can destroy and remove pathogens in their environment. Macroglia are large glial cells of the peripheral nervous system that specialize in phagocytosis; SCs are a type of macroglial cell that provides myelination to axons in the peripheral nervous system remove cellular debris through phagocytosis to promote regeneration of nerves (Haack and Hynes, 2001; Gardiner, 2011). Although they can be supportive for neuron regeneration, activated SCs assume many cellular responsibilities, some that are detrimental to nerves. Following nerve injury, SCs release a number of pro-inflammatory cytokines (such as IL-1β) that contribute to the neuro-inflammatory response (Shamash et al., 2002; Tofaris et al., 2002). Similar to SCs, monocytes also demonstrate cytotoxic and cell protective properties. Monocytes can also transform into M1 and M2 tissue macrophages. While M1 macrophages of the classical pathway secrete pro-inflammatory cytokines (such as IL-1β), monocytes that are primed by the alternative pathway (Th-2) or M2 macrophages release anti-inflammatory cytokines that promote cell survival and regeneration and express Arginase 1 (Arg1) (Martinez et al., 2008; Stout, 2010; Ydens et al., 2012). Therefore, macrophage and SC activity can be indirectly studied using markers for IL-1β and Arg1.

**Figure 7:**
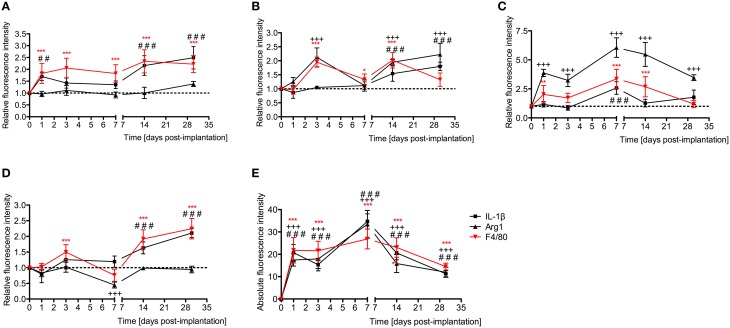
**Time-course immunofluorescence analysis for IL-1β, Arg1, and presence of macrophages/microglia (F4/80 positive cells) levels in different areas of the implanted cochleae. (A)** Lateral wall tissues (marked as SL, spiral ligament in the immunofluorescence images), **(B)** organ of Corti (OC, see Figure 8), **(C)** spiral ganglion (SG), **(D)** cochlear nerve, and **(E)** wound site (asterisk in Figure 8). ImageJ was used to analyze the images, histograms of red and green channels for each region of interest i.e., lateral wall, organ of Corti, spiral ganglion, cochlear nerve, and wound site images were recorded. *N* = 8 each time point, Two-Way ANOVA followed by Bonferroni posttests was used to analyze the relative and absolute fluorescence intensities. ^***^p < 0.001, ^**^p < 0.01, ^*^p < 0.05, +++*p* < 0.001, and ###*p* < 0.001, ##*p* < 0.01 represent the *p* values for F4/80, Arg1 and IL-1β, respectively.

**Figure 8:**
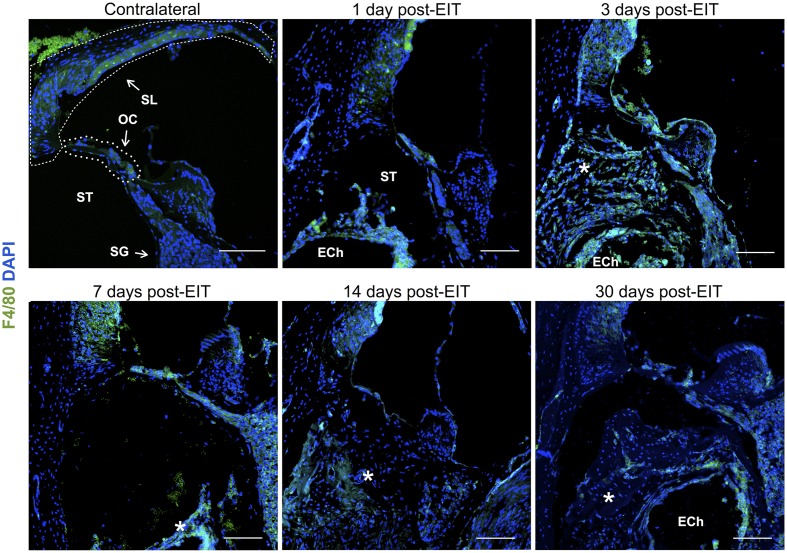
**Immunofluorescence images for F4/80 (monocytes/macrophages/microglia, green) and nuclei (blue)**. Representative micrographs of cross sections correspond to a contralateral cochlea (bottom), and cochleae at 1, 3, 7, 14, and 30 days post-implantation. A heavy influx of monocytes/macrophages/microglia can be observed at 3 days in the newly formed tissue en sheathing electrode analog as well as in the lateral wall, organ of Corti, and to a lessen extend to the spiral ganglion area. The sections were observed under a confocal microscope. Images of different areas were acquired and stitched together afterwards. Annotations for the different structures of the cochlea are in the contralateral photograph. Spiral ligament, SL; organ of Corti, OC; Scala tympanica, ST; Spiral ganglion, SG. In the implanted cochleae: electrode analog channel, ECh; an asterisk marks hyperproliferative tissue in the Scala tympanica. Four to five specimens were analyzed for each time point. Scale bars = 100 μm.

**Figure 9:**
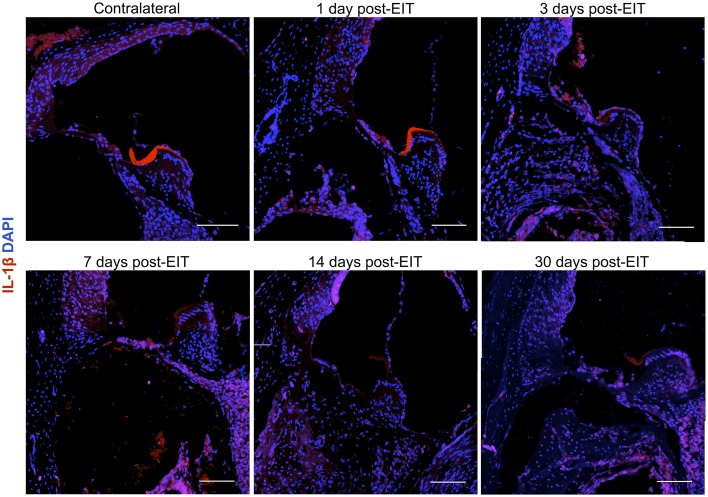
**Immunofluorescence images for interleukin-1β (IL-1β, red), and nuclei (blue)**. Representative micrographs of cross sections correspond to a contralateral cochlea (bottom), and cochleae at 1, 3, 7, 14, and 30 days post-implantation. A strong red signal at 7 days post-implantation, especially in the spiral ganglion area and wound site, reveals a severe neuro-inflammatory response. The sections were observed under a confocal microscope. Images of different areas were acquired and stitched together afterwards. Four to five specimens were analyzed for each time point. Scale bars = 100 μm.

**Figure 10:**
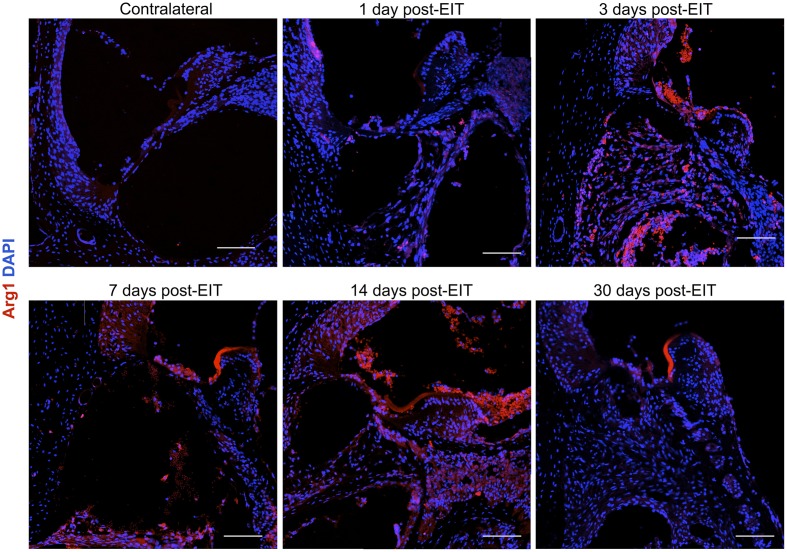
**Immunofluorescence images for Arginase I (Arg1, red) and nuclei (blue)**. Representative micrographs of cross sections are from a contralateral cochlea (bottom), and cochleae at 1, 3, 7, 14, and 30 days post-implantation. Note that red blood cells are accumulated over the organ of Corti area, these cells are auto-fluorescent due to hemoglobin fluorescence. An increase in Arg1 levels at 7 days post- implantation, especially in the spiral ganglion area and wound site, indicates healing process. The sections were observed under a confocal microscope. Images of different areas were acquired and stitched together afterwards. Four to five specimens were analyzed for each time point. Scale bars = 100 μm.

Relative fluorescence intensities (Figure 7) for IL-1β (a pro-inflammatory cytokine, indirect marker used for the classical activation pathway of M1 macrophages and SCs), Arg1 (indirect marker for the alternative pathway activated M2 macrophages), and F4/80 (marker for monocytes, macrophages and microglia) were calculated and normalized as described in the previous section. Immunofluorescence images at days 1, 3, 7, 14, and 30 post-implantation are shown for F4/80 (Figure 8), IL-1β (Figure 9), and Arg1 (Figure 10). Progressive increases in monocyte/macrophage infiltration (F4/80) and IL-1β production were seen in the lateral wall tissues over time, reaching maximum levels at 14 and 30 days post-implantation. In the OC, the IL-1β levels increased on day 14 and then remained stable until day 30. Interestingly, monocyte/macrophage infiltration and the levels of Arg1 showed a biphasic pattern of expression with one peak at 3 days and the second at 14 days. After 1 month post-implantation, the levels of Arg1 remained higher than IL-1β and the intensity of F4/80 staining for monocytes/macrophages/microglia was reduced. Results from the spiral ganglion area and wound site (area of monofilament insertion) were similar, i.e., Arg1 levels and monocyte/macrophage /microglia invasion rose rapidly at 1 day post-implantation with maximum levels detected at 7 days post-implantation. In the spiral ganglion, the levels of Arg1 predominated over IL-1β levels, peaking at day 7, indicating involvement of M2 macrophages. Expression of IL-1β and Arg1 in the wound site (area of electrode insertion) overlapped at all times, suggesting that both M1 and M2 macrophages are present. In the cochlear nerve, there is a progressive increase in monocytes/macrophages infiltration and IL-1β expression, beginning at post-implantation day 3.

### Excessive deposition of fibrotic tissue after an electrode analog insertion trauma

**Figure 11:**
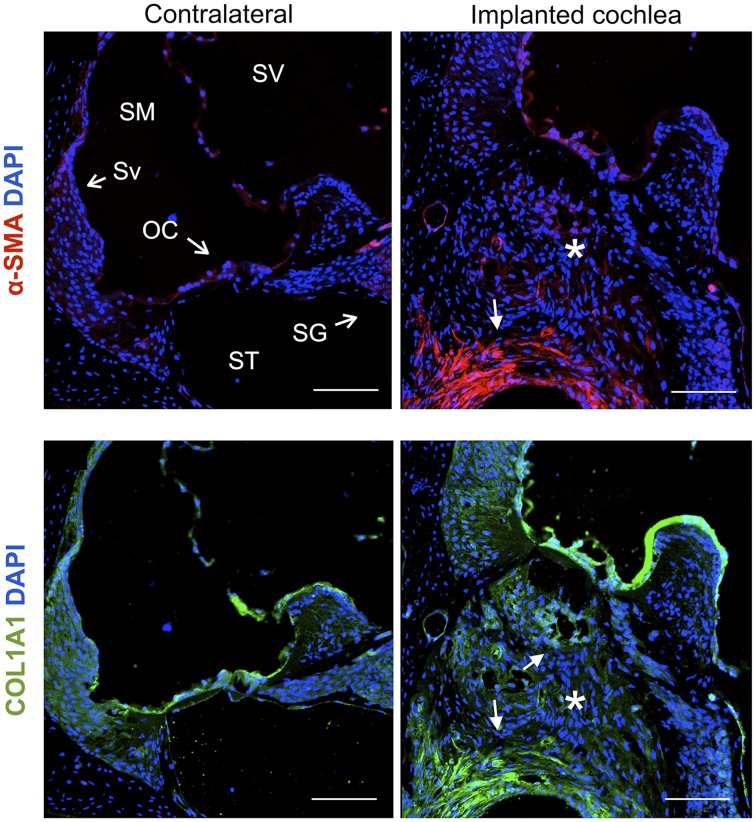
**Immunofluorescence images for the myofibroblast marker α-smooth muscle actin (α-SMA, red), Collagen type 1A (COL1A1, green) and nuclei (blue)**. Representative micrographs of cross sections correspond to a contralateral cochlea and a cochlea implanted for 1 month. Cells from the fibrotic tissue enclosing the electrode analog stain positive for α-SMA and COL1A1, asterisks indicate the presence of fibrotic tissue. The sections were observed under a confocal microscope. Images of different areas were acquired and stitched together afterwards. Scale bars = 100 μm.

Cochleae implanted with a monofilament were processed with antibodies for α-smooth muscle actin (myofibroblast marker) and Collagen type 1A 30 days after implantation and compared to contralateral control cochleae. The fibrous tissue that formed around the electrode array (O-shaped ring) demonstrated presence of myofibroblasts and expression of Collagen type 1A, while unoperated cochleae did not demonstrate any staining for these markers (Figure 11).

**Figure 12:**
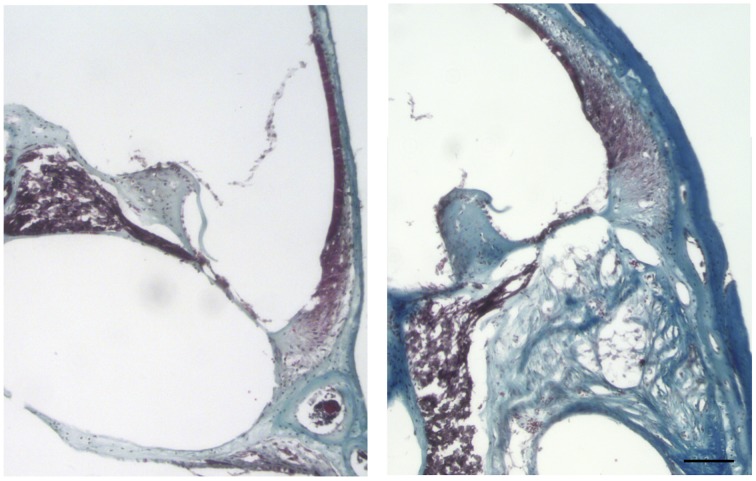
**Masson's trichrome staining for the presence of scar tissue**. Representative micrographs of section of a contralateral non-implanted cochleae on the left, section of implanted cochlea on the right. Collagen fibers stained blue, nuclei stained black and cytoplasm and erythrocytes stained red. Images of different areas were acquired and stitched together afterwards. Four contralateral and four implanted cochleae were used. Scale bar = 100 μm.

Cochleae from adult mice that received unilateral cochlear implantation *in vivo* were harvested 30 days after implantation, stained with Masson's trichrome and examined for the presence of scar tissue (Figure 12). Implanted cochleae demonstrated blue staining for collagen fibers in the scala tympani (the area of the monofilament insertion). Sections obtained from contralateral unoperated cochleae did not demonstrate blue staining for collagen. The magnitude of the scar thickness can be appreciated in Figure 12.

## Discussion

Cochlear implantation can restore hearing in patients with significant HL by electrically stimulating the neurons of SG. Preservation of residual auditory HCs and hearing and reducing injury to SGNs during and after implantation can improve hearing outcomes with electrical-acoustic stimulation (Mowry et al., 2012). However, CI surgery initiates a strong inflammatory response in the cochlea that can promote loss of SGNs and remaining auditory HCs that are crucial for hearing perception. In addition, the inflammatory response that is produced following cochlear implantation promotes fibrotic tissue deposition around the electrode array, which can also impair electrode impedance and post-implantation hearing outcomes (Hughes et al., 2001; Choi and Oghalai, 2005; Jia et al., 2011; Wolfe et al., 2013; Mosca et al., 2014). Removal of cochlear fibrosis is nearly impossible without causing significant trauma in the inner ear that can promote more inflammation and fibrotic deposition. In cases of CI device failure and need for explantation and re-implantation, intracochlear osteoneogenesis and excessive fibrosis make CI electrode insertion extremely challenging and sometimes impossible (Côté et al., 2007). Descriptions of the trauma caused by electrode insertion and associated fibrosis and new bone formation in human temporal bones can be found in the literature (Eshraghi et al., 2003; Fayad et al., 2009). The basal portion of the cochlea is most affected by EIT induced fibrosis and osteoneogenesis. The wound healing response that leads to fibrosis and new bone formation has not been well characterized following cochlear implantation. By understanding the pathophysiologic mechanisms of EIT and wound healing in the cochlea, different therapeutic strategies can be investigated to modulate fibrosis in the inner ear and prevent loss of SGNs and residual auditory HCs following cochlear implantation.

Wound healing in peripheral nerve demonstrates similarities to cutaneous tissues (Mutsaers et al., 1997; Velnar et al., 2009; Stout, 2010; Ydens et al., 2012). In injured peripheral nerve, a strong inflammatory and proliferative response occurs at the site of injury and is followed by a late remodeling phase. SCs of injured nerve rapidly release a number of pro-inflammatory cytokines, chemokines, and cell adhesion molecules that promote recruitment of inflammatory cells to the wounded area (Shamash et al., 2002; Tofaris et al., 2002). Monocytes are recruited to the injured nerve, where they transform into M1 and M2 macrophages. M1 macrophages have debride bacteria, damaged tissues, and cellular debris and release reactive radicals and pro-inflammatory cytokines (such as TNF-α and IL-1β) that are toxic to surrounding tissue during the acute and chronic inflammation. M2 macrophages secrete anti-inflammatory cytokines and factors important for nerve regeneration and tissue remodeling (Martinez et al., 2008; Stout, 2010). Both resident activated macrophages and SCs contribute to myelin down-regulation and phagocytosis (Romand and Romand, 1990). Phagocytosis, however, can be a double-edged sword in peripheral nerve injury. Although removal of apoptotic cells and debris is thought to be part of a homeostatic mechanism to prevent or delay cell death signals from spreading, phagocytosis of myelin and damaged SGNs can adversely affect the propagation of action potentials critical to auditory processing (Hurley et al., 2007).

Peripheral nerve injury can also lead to inflammation and injury more proximally. Similar to macrophages (Stout, 2010) and microglia from the central nervous system, astrocytes (the most abundant macroglial cell of the central nervous system) can exhibit polarized phenotypes induced by either classical or alternative activation in response to peripheral nerve injury (Reichert et al., 1994; de Waele et al., 1996). Reactive astrocytes have been found within the cochlear nucleus following a labyrinthectomy (Jang et al., 2013).

These classical activated macro-glia cells show neurotoxic effects and express *Il1b, Inos, Tnfa*, and *Cxcl10* genes. M1 macrophages and SCs of the classical pathway express pro-inflammatory cytokines such as IL-1β and M2 macrophages and astrocytes (activated with IL-4 and IL-13) of the alternative pathway express other regenerative and anti-apoptotic factors such as *Il10, Arg1, Mrc1, Il1ra, Fizz1*, and *Ym1*. (Perry and Gordon, 1988; Haack and Hynes, 2001; Vogelezang et al., 2001; Martinez et al., 2008; Stout, 2010; Ydens et al., 2012).

Subsequently, a proliferative phase predominates after the initial inflammatory phase of wound healing in nerve injury. Fibroblasts and activated macrophages produce large amounts of matrix proteins (fibrins and collagens) creating an extracellular matrix (ECM) that acts as a network where cell-cell (through cadherins and different cell adhesion molecules) and cell-matrix (through integrins) interactions promote migration, growth, and cellular differentiation (e.g., myofibroblast formation) (de Waele et al., 1996; Stout, 2010; Ydens et al., 2012). Fibronectin (i.e., a component of the ECM) is rapidly upregulated upon injury to peripheral nerves either by fibroblasts or endothelial cells. De-differentiated SCs in injured nerve express integrin α4β1, a receptor protein that binds fibronectin and promotes axonal regeneration and SC proliferation (Jessen and Mirsky, 1999, 2002; Haack and Hynes, 2001; Vogelezang et al., 2001; Gardiner, 2011). Although integrin α4β plays a role in nerve regeneration, the expression of α4 integrin can also mediate influx of immune cells to the injured nerve through VCAM1 binding, propagating the inflammatory response (Rose et al., 2002). After proliferation, remodeling occurs at the injured nerve, inflammation and angiogenesis declines, and scarring matures.

The wound healing process that occurs in the cochlea following EIT parallels descriptions in peripheral nerve injury. A robust inflammatory response occurs following EIT. Injured cochlear tissues express a number of pro-inflammatory factors and chemoattractants such as chemokine *Ccl2*, cell adhesion molecules *Sele* and *Vcam1*, and growth factor *Tgfb1* 3 days after EIT *in vitro* (Figure 3). Cultured monocytes migrate to the site of cochlear injury and express other pro-inflammatory cytokines, chemokines, and factors at 3 days *in vitro* (*Ccl2, Sell, Icam1*, and *Tgfb1*; Figures 1–3). In our hands, DXM did not affect the number of interactions between leukocytes and leukocyte-tissue cells, however a decrease in the duration of these interactions was observed. Similarly, other authors (Mancuso et al., 1995; Tailor et al., 1997) have already reported that DXM affects selectively the leucocyte emigration process, but not the rolling or adhesion processes in response to chemoattractants onto the microvascular tissues of the rat mesentery. Therefore, a reduction in the time of interaction may affect the leukocytes trans-endothelial emigration. Particularly in the OC, spiral ganglia, and site of EIT *in vivo*, monocytes transform into M1 macrophages that secrete other inflammatory factors (such as *IL-1ß)* and M2 macrophages that express regenerative and anti-apoptotic factors (such as *Arg1*; Figures 6–8). Increased *Arg1* expression in the spiral ganglia may in part be due to infiltrating M2 or SCs activated by the alternative pathway, promoting nerve regeneration (Reichert et al., 1994; Martinez et al., 2008; Stout, 2010; Ydens et al., 2012; Jang et al., 2013). The lateral wall, OC, spiral ganglia, and site of EIT all express integrin 4α (receptors for fibronectin important nerve regeneration and cell-matrix interactions) during the early wound healing process (Figures 4, 5) that persists in the OC, spiral ganglia, and wound bed 30 days after implantation *in vivo*. Fibroblasts and differentiated myofibroblasts proliferate in the cochlea after EIT injury and new collagen is deposited in the wound bed around the electrode analog *in vivo* (Figure 9) (Van De Water et al., 2013). F-actin was up regulated in the area of a monofilament insertion in mouse cochleae *in vitro*, suggesting there is reorganization of the actin skeleton and remodeling of the ECM (Figures 4, 6). Scar forms and matures around the electrode array in the cochlea during the remodeling phase of wound healing (Figure 12). Macrophage activity still persists at high levels in the wound bed (site of EIT) at 30 days *in vivo*, suggesting an atypical chronic inflammatory response and a possible immune response against a foreign body (the electrode analog).

Increased gene expression for leukocyte chemoattractants (*Ccl3* and *Ccl12*), pro-angiogenesis factors (*Vegfa, Hgf*), integrin subunits important for collagen deposition (*Itga1, Itga2, Itga3)*, enzymes responsible for ECM remodeling (*Mmp2, Mmp3, Mmp13, Mmp14, Plau, Serpine1, Serpinh1, Timp1, Timp2*) (La Fleur et al., 1996), components of the ECM (*Acta2, Col1a2, Col3a1)*, and mediators of ECM cross-linking and interactions (*Lox, Thbs2*) were also demonstrated in cochlear tissue following EIT *in vivo*, supporting histologic findings of wound healing in the cochlea (Table 1). *Tgfb3* and its receptor *Tgfbr2*, which are associated with scar-less wound healing, were down-regulated in EIT cochlear tissues, representing a possible therapeutic target for prevention of fibrotic scar in future studies.

In summary, cochlear implantation promotes a wound healing response in the cochlea characterized by an inflammatory phase (i.e., expression of pro-inflammatory cytokines, chemokines, and chemoattractants, leukocyte infiltration, and macrophage activation), a proliferative phase (i.e., angiogenesis, fibroblast and differentiated myofibroblast proliferation, collagen deposition, synthesis of ECM, and scar formation), and a remodeling phase (i.e., turnover of the ECM and maturation of the scar). How trauma and inflammation from EIT can initiate loss of auditory HCs and SGNs is well-described in the literature (Roehm and Hansen, 2005; Bas et al., 2012b). However, the molecular and cellular mechanisms involved in the proliferative and remodeling phases following EIT are not entirely known. The results of this study confirm key events in the inflammatory process following EIT and offer a detailed description of the proliferative and remodeling cascades of wound healing in the cochlea. A chronic inflammatory response characterized by macrophage activity is evidenced long after initial cochlear trauma and likely represents a foreign body reaction to the electrode analog (nylon filament) and a source of pro-inflammatory factors that can be detrimental to remaining HCs and viable SGNs long-term. Furthermore, fibrotic deposition in the cochlea following EIT was also confirmed and associated signaling cascades were depicted in this study. By understanding these pro-inflammatory, proliferative, and remodeling phases of wound healing in the cochlea following EIT, therapeutic strategies can be developed and tested to mitigate losses of auditory HCs and SGNs from a robust inflammatory response, reduce fibrosis that can interfere with electrode impedance, and alter chronic inflammatory changes in the inner ear from a foreign body reaction in efforts to improve residual hearing preservation, electrical-acoustic hearing outcomes, and quality of life after cochlear implantation.

## Compliance with ethical standards

Ethical approval: All applicable international, national, and/or institutional guidelines for the care and use of animals were followed. All procedures performed in studies involving animals were in accordance with the US National Institutes of Health guidelines and with the approval of the University of Miami Institutional Animal Care and Use Committee.

## Author contributions

SG participated in the analysis of the data from the *in vitro* and *in vivo* section, where she was blinded to the different groups. MA participated in the processing of the adult mice cochleae for histology. CD participated in the molecular genetic studies and helped to draft the manuscript. JB helped with the design and management of the project and the draft of the manuscript. TV and AE helped to draft the manuscript and provided important inputs during the data analysis. EB conceived of the study, and participated in its design and coordination and helped to draft the manuscript. All authors read and approved the final manuscript.

## Funding

This project was funded by the American Hearing Research Foundation to EB.

### Conflict of interest statement

TV, EB, and AE received research grant support from Med El Corporation, Innsbruck, Austria. The other authors declare that the research was conducted in the absence of any commercial or financial relationships that could be construed as a potential conflict of interest.

## Supplementary material

Supplemental Movie 1 **Cell tracking of leukocytes co-cultured with undamaged control group of organ of Corti and lateral wall tissues**. Four cochlear tissue explants (i.e., organ of Corti and lateral wall tissues) were used, with a total of 3 independent replicates. Sequential images of each group were taken every 15 s for 20 min with a 20× lens in a Zeiss LSM 700/ confocal upright microscope. ImageJ with Trackmate plugin was used to make the movies. The detected cells are included in a magenta circle and the line color codes are in function of the velocity for that particular cell. A rainbow color code displays dark blue lines for slow moving cells and red for fast moving cells (3.2 μm/s). Scale bar = 100 μm.

Supplemental Movie 2 **Raw sequential images for Supplemental Movie 1**.

Supplemental Movie 3 **Cell tracking of leukocytes co-cultured with EIT group of organ of Corti and lateral wall tissues**. Four cochlear tissue explants were used, with a total of 3 independent replicates. Sequential images of each group were taken every 15 s for 20 min with a 20× lens in a Zeiss LSM 700/ confocal upright microscope. ImageJ with Trackmate plugin was used to make the movies. The detected cells are included in a magenta circle and the line color codes are in function of the velocity for that particular cell. A rainbow color code displays dark blue lines for slow moving cells and red for fast moving cells (3.2 μm/s). Scale bar = 100 μm.

Supplemental Movie 4 **Raw sequential images for Supplemental Movie 3**.

Supplemental Movie 5 **Cell tracking of leukocytes co-cultured with EIT pre-treated with dexamethasone (20 μg/ml) group of organ of Corti and lateral wall tissues**. Four cochlear tissue explants were used, with a total of 3 independent replicates. Sequential images of each group were taken every 15 s for 20 min with a 20× lens in a Zeiss LSM 700/ confocal upright microscope. ImageJ with Trackmate plugin was used to make the movies. The detected cells are included in a magenta circle and the line color codes are in function of the velocity for that particular cell. A rainbow color code displays dark blue lines for slow moving cells and red for fast moving cells (3.2 μm/s). Scale bar = 100 μm.

Supplemental Movie 6 **Raw sequential images for Supplemental Movie 5**.
